# Packaging With Different Color Bags Under Light Exposure Improves Baby Mustard (*Brassica juncea* var. *gemmifera*) Postharvest Preservation

**DOI:** 10.3389/fpls.2022.880271

**Published:** 2022-05-18

**Authors:** Peixing Lin, Hongmei Di, Jie Ma, Yating Wang, Jia Wei, Yue Jian, Zhiqing Li, Jingyi Xu, Yangxia Zheng, Huanxiu Li, Fen Zhang, Bo Sun

**Affiliations:** ^1^College of Horticulture, Sichuan Agricultural University, Chengdu, China; ^2^Bijie Institute of Agricultural Sciences, Bijie, China; ^3^Zhejiang Academy of Agricultural Sciences, Hangzhou, China

**Keywords:** baby mustard, packaging, light exposure, sensory quality, glucosinolate

## Abstract

Effect of packaging baby mustard into bags of different color under light exposure on its visual quality and the content of chlorophyll, carotenoids, and glucosinolates at 20°C was investigated. Packaging with seven color bags under light exposure prolonged the shelf life, especially green (GB), blue (BB), and transparent (TB) bags with holes, and their shelf life was 1.7, 1.6, and 1.6 times that of the control, respectively. The GB and BB treatments delayed the deterioration of the sensory quality in baby mustard during storage. The BB and TB treatments not only increased chlorophyll and carotenoids content in baby mustard during storage but also enhanced the accumulation of glucosinolates by inhibiting their degradation, especially the BB treatment. Overall, the results demonstrate that the BB treatment is a promising technique for maintaining the postharvest quality of baby mustard.

## Introduction

Baby mustard, a variant of stem mustard, is widely consumed. Like other crucifers, baby mustard is rich in glucosinolates (Sun et al., 2018). Glucosinolates are important secondary metabolites in *Brassica* vegetables (Wang et al., 2019). According to the source of side-chain amino acids, glucosinolates can be grouped into aliphatic, indolic, and aromatic glucosinolates (Wang et al., 2019; Casajús et al., 2021). More recently glucosinolates and their breakdown products have received increased attention because they are related to reducing the risk of certain types of chronic diseases (Ragusa et al., 2017; Branca et al., 2018; Managa et al., 2019). Epidemiological studies suggest that a diet rich in *Brassica* vegetables can decrease the risk of several types of cancers, including prostate and breast cancers (Wang et al., 2021). Glucosinolates also play a role in the flavor and taste of *Brassica* vegetables (Miao et al., 2021). However, baby mustard is susceptible to the development of dehydration, browning, and the loss of bioactive substances during storage (Sun et al., 2020, 2021). In addition, the degradation of chlorophyll in baby mustard can make the vegetable browning or yellowing (Sun et al., 2021). There is thus a need to develop effective postharvest techniques to maintain postharvest quality in baby mustard at ambient storage temperature.

Several novel packing materials have been applied for maintaining the quality or inhibiting the decay in postharvest agronomic fruit or vegetable (Wang et al., 2010). Several studies have demonstrated that appropriate packaging treatment can prolong the shelf life of various vegetables and sustain, and even improve, their quality (Jia et al., 2009; Pinela et al., 2016; Guo et al., 2019). Packaging broccoli florets with polyethylene film without holes is a promising technique for sustaining their sensory quality and glucosinolates content (Jia et al., 2009). Modified-atmosphere packaging can maintain the freshness of postharvest lettuce (Guo et al., 2019). Similar results have also been obtained for cherry tomatoes (D’Aquino et al., 2016), *Toona sinensis* (Lin et al., 2019), and snap peas (Elwan et al., 2015). Irradiation has a beneficial influence on the sensory quality and nutritional indicators of various vegetables after harvest, and it has been successfully applied to numerous vegetables, such as Brussels sprouts (Hasperué et al., 2016), pak choi (Song et al., 2020; Zhou et al., 2020), purple kale (Bárcena et al., 2019), *Ocimum basilicum* leaves (Costa et al., 2013), and broccoli (Ma et al., 2014; Jin et al., 2015; Favre et al., 2018). Our previous study has shown that light treatment (36 μmol m^–2^ s^–1^) can prolong the shelf life and ensure the visual and nutritional qualities in baby mustard (Sun et al., 2021). The factors that affect the effectiveness of light treatment during postharvest storage include light quality and light intensity (Song et al., 2020). Optimal light quality and light intensity conditions vary among species. Broccoli samples stored under green (24 μmol m^–2^ s^–1^, 522 nm), red (66 μmol m^–2^ s^–1^, 625 nm), white (40 μmol m^–2^ s^–1^), and yellow (27 μmol m^–2^ s^–1^, 587 nm) light have a longer shelf life of 5 days than those treated with dark and blue (21 μmol m^–2^ s^–1^, 467 nm) light (Loi et al., 2019). However, blue light (main emission spectrum 400 to 550 nm) preserves the sensory quality of white asparagus for a longer period than white light (Sanz et al., 2009). In pak choi, 10 μmol m^–2^ s^–1^ light treatment was found to be effective in suppressing senescence and maintaining quality (Bárcena et al., 2019), whereas higher light (24 μmol m^–2^ s^–1^) treatment was shown to be best for suppressing senescence in broccoli (Zhan et al., 2012). Since the optimal light treatment conditions vary among vegetable crops, studies are needed to determine the optimal light quality and light intensity treatment conditions for the postharvest storage of different vegetable crops.

Research examining the influence of differences in light quality on the storage of postharvest vegetables is often conducted via the application of light-emitting diode (LED). However, most LED irradiation processing devices have only been applied in the laboratory; they have not yet been used in storage, transportation, and sales terminals such as cold storage rooms, supermarket shelves, and refrigerators. The color of bags used for packaging can modify the radiation spectrum reaching the surface of vegetables. Therefore, packaging with bags of different color coupled with light exposure can provide the benefits of packaging as well as optimize the light quality and light intensity treatment for the postharvest storage of vegetables. Compared with light quality irradiation, packaging with different color bags coupled with light exposure is more convenient and cost-effective. The purpose of this study was to explore the effect of storing baby mustard in bags of various color under light exposure on their sensory quality and content of chlorophyll, carotenoids, and glucosinolates and identify the optimal color of the bags.

## Materials and Methods

### Plant Materials

Baby mustard (*Brassica juncea* var. *gemmifera* cv. Linjiang-Ercai) was planted in an open field at a local farm in Chengdu City, China. Heads with a uniform size and an absence of external damage were collected and transported to the laboratory within 2 h of harvest. The lateral buds were removed from the heads of baby mustard and assigned randomly to various treatment groups.

### Packaging and Storage Treatments

In experiment I, baby mustard was packaged into bags of seven different colors with and without holes to evaluate the optimal packaging conditions for delaying senescence under light exposure. The lateral buds were randomly packaged into transparent (TB), white (WB), red (RB), yellow (YB), green (GB), blue (BB), and black (DB) bags (17 cm × 25 cm, 70 μm thickness), and the packaging of each color bags either had four holes on each side of the bag (6 mm in diameter) or did not have holes. The O_2_ and CO_2_ transmission rates of polyethylene bags are 7.0 × 10^–7^L m^–2^s^–1^ and 2.4 × 10^–6^L m^–2^s^–1^ at 25°C and standard pressure, respectively. The lateral buds were stored in an incubator at 20°C, 75% relative humidity, white light, 12-h photoperiod, and 37 μmol m^–2^ s^–1^ light intensity. Non-wrapped baby mustard was used as the control and stored in darkness under the same other conditions.

Experiment II was designed based on the results of experiment I. The top three treatments in terms of the shelf life were selected to further explore quality evolution in baby mustard. The lateral buds were randomly packaged into transparent, green, and blue bags, and holes were punched. The conditions of the control and other treatments were the same as those in experiment I. Pre-storage (0) and 2, 4, and 6 days after storage, sensory quality, weight loss, chlorophyll, carotenoids, glucosinolates, and glucosinolate metabolism-related gene expression were evaluated to clarify the effect of packaging with different color bags under light exposure on the postharvest quality of baby mustard. Four replicates were used per sampling period of each treatment, and one bag (approximately 300 g) was one replicate.

### Shelf Life and Sensory Quality Evaluation

The lateral buds were considered to have reached the end of their shelf life when they became soft, shrunken, and exhibited browning (Sun et al., 2021). Sensory attributes, including color, odor, texture, and acceptance, were quantified on a 5-point scale. Where 5 = full characteristic of the product, 3 = moderate, and 1 = no characteristic. A score below 3 for any of these sensory attributes was deemed to be the end of shelf life (Lin et al., 2021).

### Light Transmittance Rate and Spectral Composition of Transmission Light

The light transmittance rate and the spectral composition of transmission light of different color bags were measured by a portable handheld spectrometer. The light transmittance rate (%) was calculated by the formula I_*I*_/I_*o*_ × 100, where I_*o*_ is the light intensity outside the bag, and I_*I*_ is that inside the bag.

### Weight Loss

Weight loss (%) was calculated by the formula (W_*x*_ - W_0_)/W_0_ × 100, where W_0_ is the weight at 0 day, and W_*x*_ is the weight at a certain day after storage (Sun et al., 2021).

### Chlorophyll and Carotenoid Content

Freeze-dried samples (200 mg) were extracted with 25 mL acetone. The samples were sonicated for 20 min and centrifuged. The supernatant was filtered and analyzed by high-performance liquid chromatography (HPLC). HPLC analysis of chlorophyll and carotenoid was carried out using an Agilent 1260 instrument with a variable wavelength (VWD) detector. Samples (10 μL) were separated at 30°C on a Waters C18 column (150 × 3.9 mm) using isopropanol and 80% acetonitrile-water at a flow rate of 0.5 mL min^–1^. Absorbance was detected at 448 and 428 nm. Result of chlorophyll and carotenoid content was expressed as g kg^–1^ of dry weight (Sun et al., 2021).

### Glucosinolate Composition and Content

Freeze-dried samples (100 mg) were boiled in 5 mL water for 10 min. The supernatant was collected and applied to a DEAE-Sephadex A-25 column. The glucosinolates were converted into their desulfo analogs by overnight treated with 100 μL of 0.1% aryl sulfatase, and the desulphoglucosinolates were eluted with 1 mL water. Desulphoglucosinolates was analyzed by HPLC. HPLC analysis of desulphoglucosinolates was carried out using an Agilent 1260 HPLC instrument equipped with a VWD detector. Samples were separated at 30°C on a Waters Spherisorb C18 column (250 × 4.6 mm) using acetonitrile and water at a flow rate of 1.0 mL min^–1^. Absorbance was detected at 226 nm. Result of glucosinolate content was expressed as mmol kg^–1^ of dry weight (Sun et al., 2018).

### RNA Extraction and qPCR Analysis

Frozen samples were used for RNA extraction using an RNA Easy Fast Plant Tissue Kit. The qPCR reaction was performed referring to the TB Green Premix Ex Taq II kit instructions using the Bio-Rad iCycler Thermocycler. The expression profiles of baby mustard under storage with different treatments at 2 days were characterized. The relative levels of gene expression were calculated according to the formula 2^–ΔΔ*CT*^ (Livak and Schmittgen, 2001). Glucosinolate-related gene sequence data were found in the Brassica database (BRAD)^1^ under the following accession numbers: *MYB28* (BjuA032531), *MYB34* (BjuB037750), *CYP79F1* (BjuA022066), *CYP79B2* (BjuO008644), *CYP79B3* (BjuB024228), *AOP2* (BjuA031496), *TGG1* (BjuA032846), *TGG5* (BjuA045911), *PEN2* (BjuA001453), and *PEN3* (BjuB022172); β*-actin* was used as a reference gene. Sequences for the primers were listed in Supplementary Table 1.

### Statistical Analysis

Statistical analysis and correlation analysis were performed using the SPSS package program version 18. Data were analyzed using one-way ANOVAs. A time-related trajectory analysis based on a two-dimensional principal component analysis map was used to visualize temporal changes in postharvest quality among different storage treatments (Sun et al., 2021).

## Results

### Green Bags, Blue Bags, and Transparent Bags Treatments Extended the Shelf Life in Baby Mustard

**FIGURE 1:**
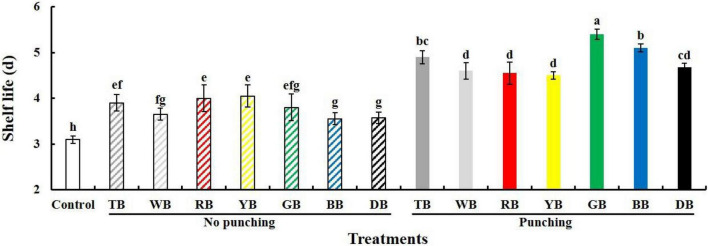
Shelf life of the lateral buds of baby mustard packed with seven color bags under light exposure during storage. TB, WB, RB, YB, GB, BB, and DB indicate treatments in which lateral buds of baby mustard were packed into transparent, white, red, yellow, green, blue, and black bags during storage, respectively. Different letters in the figure indicate statistically significant differences among treatments (*P* < 0.05).

Baby mustard deteriorates rapidly and has a short shelf life. Packaging treatments extended the shelf life of postharvest baby mustard (Figure 1). The shelf life was longer in bags with holes than without holes. The top three treatments in terms of shelf life were the GB, BB, and TB treatments with holes, and their shelf life was 1.7, 1.6, and 1.6 times compared with the control, respectively. In light of this, the GB, BB, and TB treatments with holes were used in experiment II to investigate the influence of packaging with different color bags.

### Light Transmittance Rate and Spectral Composition of Transmission Light

The light transmittance rate and spectral composition of transmission light of different color bags are shown in Supplementary Table 2. The light transmittance rate of the three color bags differed. The transparent bags had the highest light transmittance rate (97.04%), followed by the green bags (76.58%) and the blue bags (52.01%). There were also differences in the spectral composition of the transmission light of the three color bags. Transparent and blue bags had higher blue light transmittance than green bags. The transparent bags had the highest transmittance of red light, followed by green bags and blue bags. Therefore, the blue bags had the highest ratio of blue and red light of the transmission light.

### Green Bags and Blue Bags Treatments Inhibited the Deterioration of the Sensory Quality in Baby Mustard

**FIGURE 2:**
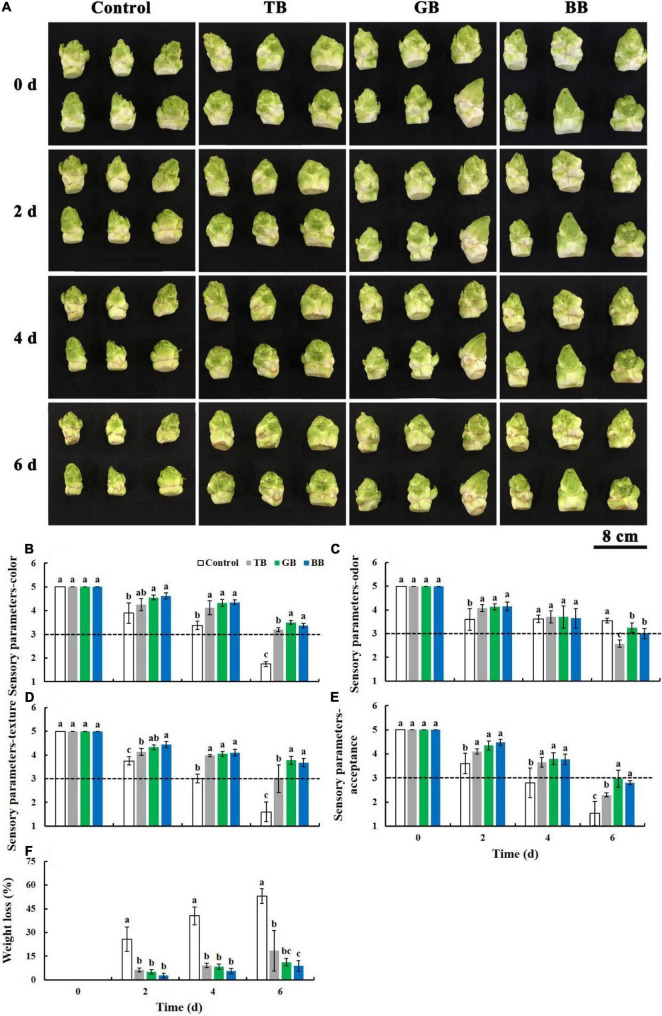
Sensory quality of the lateral buds of baby mustard packed with different color bags under light exposure during storage. **(A)** Visual external aspect of baby mustard lateral buds. **(B)** Sensory parameters-color. **(C)** Sensory parameters-odor. **(D)** Sensory parameters-texture. **(E)** Sensory parameters-acceptance. **(F)** Weight loss. TB, GB, and BB indicate treatments in which lateral buds of baby mustard were packed into transparent, green, and blue bags during storage, respectively. Different letters in the figure indicate statistically significant differences among treatments for each storage day (*P* < 0.05).

The lateral buds rapidly shriveled and browned on the peel of the control at room temperature, and packaging treatments suppressed the deterioration of the visual quality (Figure 2A). Changes in the visual quality were evaluated through four sensory parameters: color, odor, texture, and acceptance. Sensory parameter scores gradually dwindled in all treatments. The color, texture, and acceptance scores of the GB and BB treatments were higher than those of the control. The odor score of the packaging treatments was higher than that of the control at 2 days and lower than that of the control at 6 days; the odor score was higher in the GB and BB treatments than in the TB treatment (Figures 2B–E).

Weight loss under all treatments was increased during storage. All packaging treatments restrained increases in weight loss (Figure 2F). The lowest weight loss was observed in BB-treated baby mustard, which was 83.3% lower than the control at 6 days.

### Transparent Bags and Blue Bags Treatments Increased the Accumulation of Pigments in Baby Mustard

**FIGURE 3:**
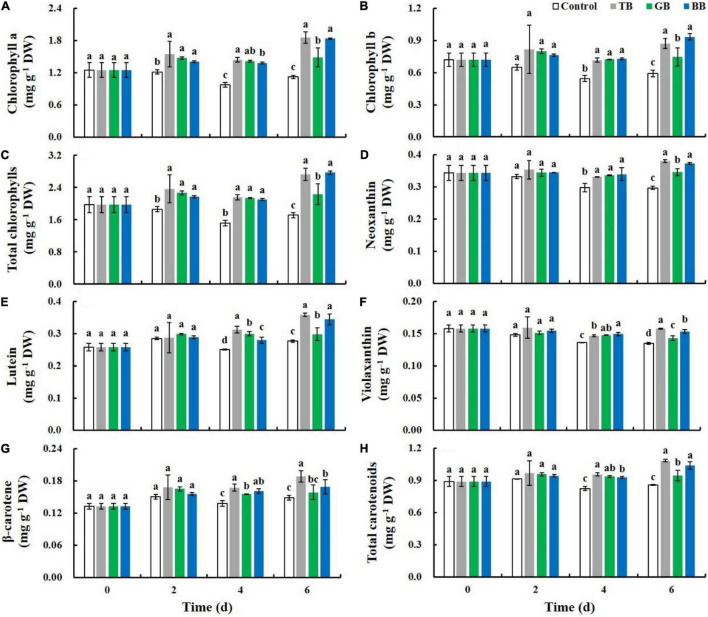
Chlorophyll and carotenoids content of the lateral buds of baby mustard packed with different color bags under light exposure during storage. **(A)** Chlorophyll a. **(B)** Chlorophyll b. **(C)** Total chlorophylls. **(D)** Neoxanthin. **(E)** Lutein. **(F)** Violaxanthin. **(G)** β-Carotene. **(H)** Total carotenoids. TB, GB, and BB indicate treatments in which lateral buds of baby mustard were packed into transparent, green, and blue bags during storage, respectively. Different letters in the figure indicate statistically significant differences among treatments for each storage day (*P* < 0.05).

The individual and total chlorophyll content in the control decreased during storage, while those of packaged baby mustard under light exposure increased. At 6 days of storage, the individual and total chlorophyll content was higher in the TB and BB treatments than in the GB treatment and the control, and the total chlorophyll content of the TB and BB treatments was 59.4 and 61.9% higher compared with the control, respectively (Figures 3A–C). After 2 days of storage, the individual and total carotenoid content was higher in the packaging treatments than in the control. At 6 days of storage, the individual and total carotenoid content was higher in the TB and BB treatments than in the GB treatment and the control; the total carotenoid content of the TB and BB treatments was 26.5 and 21.1% higher than the control, respectively (Figures 3D–H).

### Blue Bags and Transparent Bags Treatments Increased the Accumulation of Glucosinolates in Baby Mustard

**FIGURE 4:**
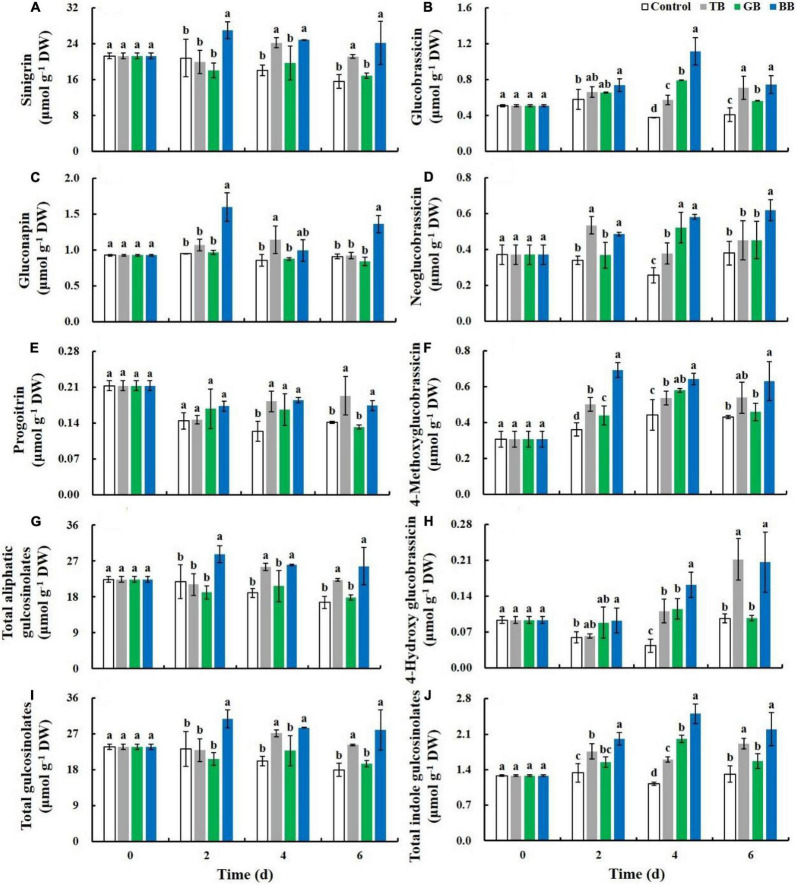
Glucosinolates content of the lateral buds of baby mustard packed with different color bags under light exposure during storage. **(A)** Sinigrin. **(B)** Glucobrassicin. **(C)** Gluconapin. **(D)** Neoglucobrassicin. **(E)** Progoitrin. **(F)** 4-Methoxyglucobrassicin. **(G)** Total aliphatic gulcosinolates. **(H)** 4-Hydroxy glucobrassicin. **(I)** Total gulcosinolates. **(J)** Total indole gulcosinolates.TB, GB, and BB indicate treatments in which lateral buds of baby mustard were packed into transparent, green, and blue bags during storage, respectively. Different letters in the figure indicate statistically significant differences among treatments for each storage day (*P* < 0.05).

During storage, the content of sinigrin, progoitrin, and total aliphatic glucosinolates decreased, and the content of gluconapin remained basically unchanged in the control. However, the BB treatment increased the accumulation of all aliphatic glucosinolates, and the total aliphatic glucosinolate content of BB-treated baby mustard was 1.5 times that of the control at 6 days (Figures 4A,C,E,G). The indole glucosinolate content fluctuated slightly during storage in the control. The BB treatment increased the accumulation of all indole glucosinolates, and the total indole glucosinolate content of BB-treated baby mustard was 1.7 times that of the control at 6 days (Figures 4B,D,F,H). Therefore, the BB treatment increased the accumulation of total glucosinolates, and the total glucosinolate content of BB-treated baby mustard was 1.6 times higher than that of the control at 6 days (Figure 4J). In addition, the TB treatment increased the accumulation of certain glucosinolates, and the total glucosinolate content of TB-treated baby mustard was 1.3 times that of the control at 6 days.

### Packaging Treatments Downregulated the Expression of Glucosinolate-Related Genes

**FIGURE 5:**
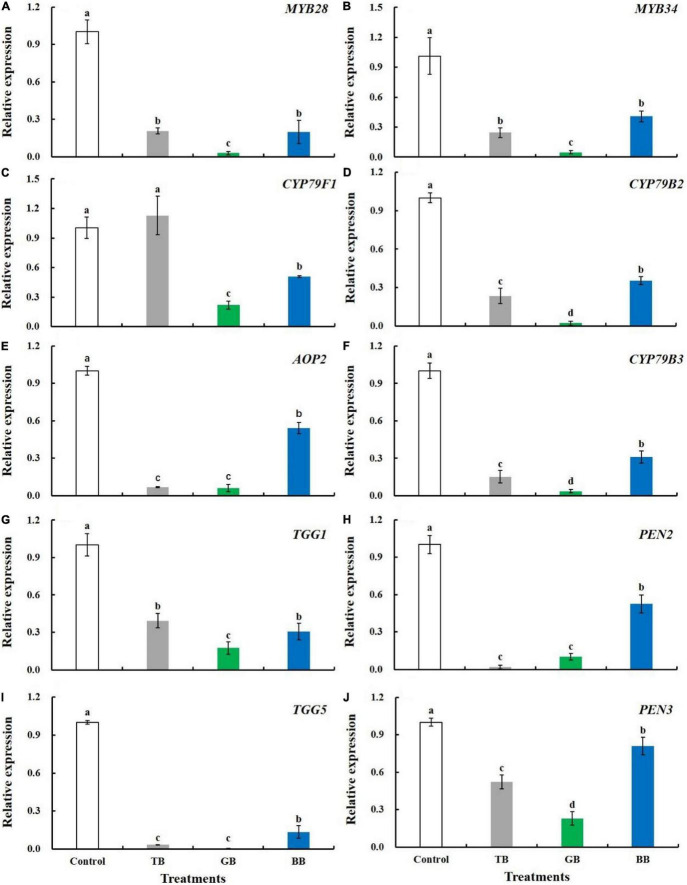
Expression levels of glucosinolate metabolism-related genes of the lateral buds of baby mustard packed with different color bags under light exposure during storage. **(A)** *MYB28*. **(B)** *MYB34*. **(C)** *CYP79F1*. **(D)** *CYP79B2*. **(E)** *AOP2*. **(F)** *CYB79B3*. **(G)** *TGG1*. **(H)** *PEN2*. **(I)** *TGG5*. **(J)** *PEN3*. TB, GB, and BB indicate treatments in which lateral buds of baby mustard were packed into transparent, green, and blue bags during storage, respectively. Different letters in the figure indicate statistically significant differences among treatments (*P* < 0.05).

The expression of all genes that regulate both the biosynthesis and degradation of glucosinolates was downregulated by packaging treatments, except for the expression of *CYP79F1* under the TB treatment (Figure 5). For genes that regulate the biosynthesis of aliphatic glucosinolates, the expression level of *MYB28* was downregulated by 79.3, 97.1, and 80.2% after the TB, GB, and BB treatments, respectively; the expression level of *CYP79F1* was downregulated by 78.3 and 49.4% after the GB and BB treatments, respectively; the expression level of *AOP2* was downregulated by 93.3, 94.0, and 45.8% after the TB, GB, and BB treatments, respectively. For genes that regulate the biosynthesis of indole glucosinolates, the expression levels of *MYB34*, *CYP79B2*, and *CYB79B3* were downregulated by the GB treatment, and the downregulation of these genes was less pronounced in the TB and BB treatments than in the GB treatment. For genes that regulate the degradation of the typical glucosinolate-myrosinase system pathway, the expression of *TGG1* was downregulated by the GB treatment, followed by the TB and BB treatments; the expression of *TGG5* was downregulated by the TB and GB treatments, followed by the BB treatment. For genes that regulate the degradation of the atypical myrosinase metabolic pathway, the expression of *PEN2* was downregulated by the TB and GB treatments; the expression of *PEN3* was downregulated by the GB treatment, followed by the TB and BB treatments.

### Shelf Life Was Positively Correlated With Glucosinolates

A correlation analysis was performed to investigate the correlations of shelf life and glucosinolates content (Table 1). During storage, shelf life was positively correlated with all glucosinolates content. Among them, shelf life was strongly positively correlated with progoitrin, glucobrassicin, 4-hydroxyglucobrassicin, and total indole glucosinolates at 2 days. Shelf life was strongly positively correlated with progoitrin, indole glucosinolates, and total glucosinolates at 4 days. Shelf life was strongly positively correlated with glucobrassicin and total indole glucosinolates at 6 days. Therefore, the correlation between shelf life and glucosinolates varied with storage time, and shelf life was strongly positively correlated with indole glucosinolates during the whole storage period.

**TABLE 1:** Correlation of shelf life and glucosinolates in the lateral buds.

	Shelf life		
	
	2 days	4 days	6 days
Sinigrin	0.05	0.60	0.54
gluconapin	0.37	0.38	0.22
Progoitrin	0.72	0.89	0.26
4-Methoxyglucobrassicin	0.58	0.87	0.53
Neoglucobrassicin	0.50	0.87	0.60
Glucobrassicin	0.80	0.74	0.76
4-Hydroxyglucobrassicin	0.73	0.86	0.42
Total aliphatic glucosinolates	0.07	0.59	0.53
Total indole glucosinolates	0.68	0.81	0.65
Total glucosinolates	0.11	0.66	0.54

### Blue Bags Treatment Was an Effective Method for Preserving the Postharvest Quality of Baby Mustard

**FIGURE 6:**
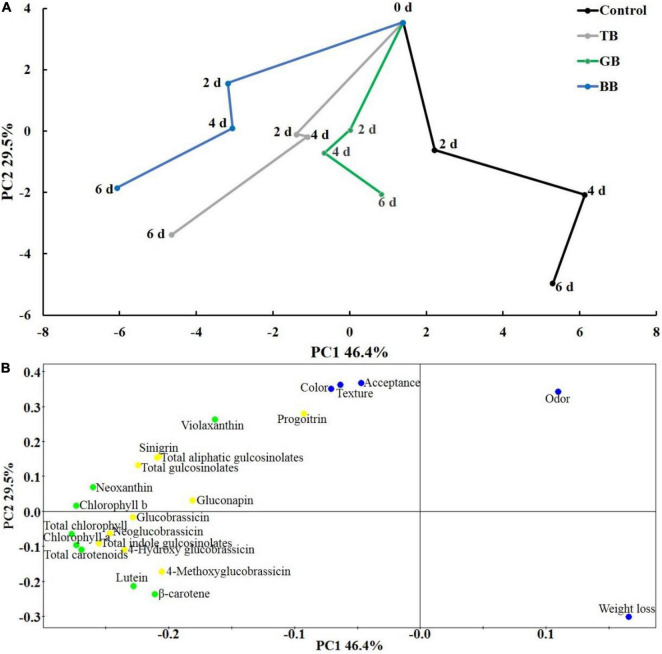
Time-related trajectory plot **(A)** and PCA loading plot **(B)**. TB, GB, and BB indicate treatments in which lateral buds of baby mustard were packed into transparent, green, and blue bags during storage, respectively.

The first component (PC1) explained 46.4% of the variation in the data, and the second component (PC2) explained 29.5% of the variation (Figure 6). Greater distance from day 0 indicated greater degrees of change in the lateral buds after harvest. Figure 6A shows that the postharvest status of the lateral buds under the control was positively correlated with PC1, and the postharvest status in the BB and TB treatments was negatively correlated with PC1. The postharvest status of the lateral buds under all treatments and the control was negatively correlated with PC2. However, the PC2 value of the control was the lowest, and the PC2 values of the GB and BB treatments were higher than those of TB treatment and the control. Figure 6B shows that PC1 was negatively correlated with chlorophyll, carotenoids, and glucosinolates; PC2 was positively correlated with the sensory parameter scores and negatively correlated with weight less. These observations indicate that the GB and BB treatments inhibited the deterioration of the sensory quality of baby mustard; the BB and TB treatments increased the accumulation of chlorophyll, carotenoids, and glucosinolates in baby mustard, especially the BB treatment. Thus, the BB treatment was an effective method for preserving the sensory quality of baby mustard after harvest and promoting the accumulation of chlorophyll, carotenoids, and glucosinolates.

## Discussion

In recent years, light treatment and packaging treatment have been used to delay the postharvest deterioration of vegetables (Jia et al., 2009; Jin et al., 2015; Hasperué et al., 2016; Guo et al., 2019; Song et al., 2020). Our previous studies of baby mustard have shown that light exposure (Sun et al., 2021) or packaging (Lin et al., 2021) alone during storage can delay postharvest senescence. In this study, we analyzed the ability of packaging baby mustard into bags of seven different colors under light exposure to delay senescence during postharvest storage. Packaging treatments extended the shelf life of postharvest baby mustard (Figure 1). This stems from the fact that the packaging treatments contribute to the formation of a spontaneous atmosphere in the storage environment, thereby reducing the respiration rate and delaying senescence (Ozturk et al., 2021). In addition, a high-humidity environment is generated inside the bag, which reduces water loss (Jia et al., 2009), thus prolonging shelf life. Similar results have also been observed in broccoli florets (Jia et al., 2009), watercress (Pinela et al., 2016), and *Toona sinensis* (Lin et al., 2019). Previous research on packaging technology has shown that the size and numbers of the holes in the film affect the content of phytochemicals and the shelf life of broccoli (Jia et al., 2009). The shelf life of baby mustard was longer when it was packaged into polyethylene bags with holes than when it was packaged into polyethylene bags without holes (Figure 1). Light can effectively increase the shelf life of postharvest vegetables, and light quality and light intensity affect the effectiveness of light treatment in previous studies (Song et al., 2020). Bags of different color can absorb or reflect incoming light, thereby controlling the radiation spectrum reaching the surface of baby mustard and thus influence the shelf life of baby mustard. The top three treatments in terms of shelf life were the GB, BB, and TB treatments with holes (Figure 1).

Sensory quality is an important factor affecting customer decisions to purchase vegetables (Lin et al., 2019). The GB, BB, and TB treatments inhibited the decrease in the color, texture, and acceptance of the sensory parameters during storage in this study (Figures 2B,D,E, 6). There may be two reasons for this result. First, light can reverse the postharvest senescence of vegetables induced or accelerated by darkness; similar results have been obtained in various vegetables, such as *Ocimum basilicum* leaves (Costa et al., 2013), broccoli (Favre et al., 2018), and purple kale (Bárcena et al., 2019). Second, packaging treatment alters the microenvironment in the bag, which reduces the respiration rate of baby mustard and thereby suppresses the deterioration of sensory quality (Ozturk et al., 2021). The GB and BB treatments had better color, texture, and acceptance scores than the TB treatment (Figures 2B,D,E, 6), which might stem from the fact that the light intensity of the blue (19.2 μmol m^–2^s^–1^) and green (28.4 μmol m^–2^s^–1^) bags was less than that of the transparent bags (36 μmol m^–2^s^–1^) (Supplementary Table 2). Previous studies have shown that below 30 μmol m^–2^s^–1^ light intensities are always used during storage, as they delay the senescence of vegetables (Büchert et al., 2011; Costa et al., 2013; Bárcena et al., 2020). 24 μmol m^–2^s^–1^ light exposure maintained the quality and extended the shelf life in broccoli (Zhan et al., 2012); 20–25 μmol m^–2^s^–1^ light treatment was reported to maintain the sensory quality of purple lettuce (Bárcena et al., 2019), which was similar to the light intensity of the blue and green bags in this study. Although the sensory parameter odor scores of the TB, GB, and BB treatments were higher than the control at 2 days, the scores of the TB, GB, and BB treatments were lower than the control at 6 days, which may stem from the fact that the packaging inhibited the interaction between the sample and the surrounding environment. This negative effect appeared gradually as the storage time extended. However, only the odor score of the TB-treated baby mustard was lower than 3 points at the end of storage. Therefore, the odor score was the main parameter limiting the sensory quality of baby mustard under the TB treatment, and this was not the main parameter limiting the sensory quality of baby mustard under the GB and BB treatments.

Weight loss and the external aspect of postharvest vegetables go hand in hand during storage (Guo et al., 2019). Studies of various vegetables such as broccoli (Paulsen et al., 2018), lettuce (Guo et al., 2019), and *Toona sinensis* (Lin et al., 2019) have shown that packaging treatment can reduce weight loss during storage. Similarly, we found that the packaging treatment inhibited the weight loss of baby mustard during storage, which may stem from the fact that packaging treatments provided a relatively closed environment for the storage of baby mustard (Jia et al., 2009; Paulsen et al., 2018) and reduced weight loss by reducing respiration and transpiration (Guo et al., 2019). Light treatment stimulates the opening of the stomata, which increases gas exchange between the plant tissue and the atmosphere inside the package (Olarte et al., 2009). The light intensity in the transparent bags was the highest, and this may have had the greatest impact on the stimulation of stomatal opening. Therefore, weight loss was higher in the TB treatment than in the GB and BB treatments. In addition, there is not an acceptable limit of weight loss for baby mustard as an absolute value. In future research, we will try to find an acceptable limit of weight loss for baby mustard as an absolute value based on the series of changes involved in the storage of baby mustard.

Changes in tissue color that accompany senescence are the key limiting factor the shelf life of vegetables on the market (Li et al., 2021). Chlorophyll and carotenoids play a decisive role in the color of lateral buds (Sun et al., 2018). Previous studies have shown that senescence involves the massive degradation of chlorophyll and carotenoids during storage (Mastropasqua et al., 2016; Gogo et al., 2017; Bárcena et al., 2020). In our study, chlorophyll and most carotenoids content decreased in the control during storage, but packaging treatments under light exposure increased the accumulation of chlorophyll and carotenoids of baby mustard during storage (Figures 3, 6). Previous studies have revealed that appropriate light treatment can increase the synthesis of photosynthetic pigments and inhibit their degradation (Hasperué et al., 2016; Bárcena et al., 2020). Light treatment can cause the accumulation of chlorophyll in cabbage (Bárcena et al., 2020) and carotenoids in radish microgreens (Xiao et al., 2014). In addition, packaging treatment can inhibit the degradation of chlorophyll and carotenoids in broccoli and lettuce (Barth and Zhuang, 1996; Guo et al., 2019). These findings indicate that packaging treatment under light exposure can increase the accumulation of chlorophyll and carotenoids during storage. The effects of the TB and BB treatments on the accumulation of chlorophyll and carotenoids in baby mustard were stronger than those of the GB treatment. Light quality regulates the metabolism of higher plants and affects their color and other qualities. The content of chlorophyll increased in cotton plantlets under blue LED light (Li et al., 2010). Transparent and blue bags have higher blue light transmittance than the green bags in our study (Supplementary Table 2). Therefore, the transmittance of different wavelengths may affect the accumulation of chlorophyll and carotenoids.

Previous studies have reported that glucosinolates content in *Brassica* vegetables can be increased by several postharvest treatments, including light treatment (Jin et al., 2015) and controlled atmosphere storage (Xu et al., 2006). In our study, the BB and TB treatments under light exposure increased the glucosinolate content in baby mustard (Figures 4, 6). This stemmed from the combined effects of light exposure and packaging treatment. Light facilitates the maintenance, and even the accumulation, of glucosinolates by maintaining the integrity of the cell structure, which inhibits the degradation of glucosinolates and promotes the synthesis of glucosinolates (Sun et al., 2021). Packaging treatment can also regulate the storage microenvironment to dwindle the respiration rate, resulting in inhibiting the degradation of glucosinolates (Jia et al., 2009). The response of phytochemical metabolism in baby mustard might depend on light intensity and quality (Jin et al., 2015). Glucosinolate content in postharvest vegetables are determined by the balance between their degradation and biosynthesis (Sun et al., 2021). Packaging treatment under light treatment inhibited the expression of genes that regulate glucosinolate biosynthesis and degradation. Therefore, the levels of glucosinolates in baby mustard may stem from the simultaneous inhibition of glucosinolate biosynthesis and degradation. The strength of the inhibition of the degradation of glucosinolates was greater than the inhibition of the biosynthesis of glucosinolates in baby mustard. To further explore the molecular mechanism of glucosinolate accumulation, we will analyze changes in glucosinolate metabolism-related genes expression levels throughout the storage period in future research.

Generally, consumers cannot directly determine glucosinolates content in vegetables. However, our results showed that shelf life had a positive correlation with glucosinolates content, especially indole glucosinolates (Table 1). The close relationship between shelf life and glucosinolates can guide consumers in the purchase of products containing higher concentrations of glucosinolate.

## Conclusion

The GB, BB, and TB treatments with light exposure extended the shelf life of baby mustard. The GB and BB treatments inhibited the deterioration of the sensory quality. The BB and TB treatments increased the accumulation of chlorophyll, carotenoids, and glucosinolates in baby mustard, especially the BB treatment. Therefore, the BB treatment could be an effective technique for preserving the sensory quality of baby mustard postharvest as well as increasing the accumulation of chlorophyll, carotenoids, and glucosinolates.

## Data Availability Statement

The original contributions presented in the study are included in the article/Supplementary Material, further inquiries can be directed to the corresponding author/s.

## Author Contributions

PL: investigation, writing–original draft preparation, and data curation. HD and JM: investigation and writing–original draft preparation. YW and JX: data curation. JW, YJ, and ZL: investigation. YZ and HL: funding acquisition and data curation. FZ and BS: funding acquisition, conceptualization, and writing–review and editing. All authors: review and editing.

## Conflict of Interest

The authors declare that the research was conducted in the absence of any commercial or financial relationships that could be construed as a potential conflict of interest.

## Publisher’s Note

All claims expressed in this article are solely those of the authors and do not necessarily represent those of their affiliated organizations, or those of the publisher, the editors and the reviewers. Any product that may be evaluated in this article, or claim that may be made by its manufacturer, is not guaranteed or endorsed by the publisher.

## Footnotes

1
http://brassicadb.org/brad/
