# Supplementary material for: Packaging With Different Color Bags Under Light Exposure Improves Baby Mustard (Brassica juncea var. gemmifera) Postharvest Preservation
Source: Front Plant Sci. 2022 May 18;13:880271. doi: 10.3389/fpls.2022.880271 (PMC9158537; doi:10.3389/fpls.2022.880271)
Supplement: Supplementary file 1 [file Data_Sheet_1.docx]

**Supplementary Table 1 Primer used in this study**

| Gene | Forward | Reversed |
| --- | --- | --- |
| *ACTIN* | CGGTTCCTCTCGTACTAGGTTGA | CCGTCGTGAGACAGGTTAGTTTT |
| *MYB28* | AAAGAGGCGAGTTTAGTTCAGA | ATCAAACGCTTCTTGAGATGTG |
| *MYB34* | CAGAAAGGTATCGATCCAGTCA | TTTGAATCGACCGAGTATTTGC |
| *CYP79F1* | CAAGCGGAGAAAGATCATCTTG | GGATTGTTGTAACTGCGAACTT |
| *CYP79B2* | CGGTCACGAGAAGATTATGAGA | GCCATTACAAGCTCCTTAATGG |
| *CYP79B3* | ATGTCCTAAGATAGCACGTGAG | TATCTTTTGGGCGTAAGTGAGT |
| *AOP2* | ATGAATGGTAGACTGTCTCGTC | GCACAATAAGCGTGAAGAGTAG |
| *TGG1* | CGACAAGCTTTGTATCAAGACC | AAGGACTCATCATTAGTCGTGG |
| *TGG5* | GATATCCAGAGAAAGTGCCAGA | CTGTATGCTTGAGCCTTCATTC |
| *PEN2* | CGTCGATTTCTTCCATCGTTAC | GGAGTGATCTCATTGGCTAGAA |
| *PEN3* | TGTCTTCGACAAGTACTCAGTC | GCGATGATGATTATCTGAACCG |

**Supplementary Table 2 Light transmittance rate and spectral composition of transmission light**

| Treatment | Light transmittance rate (%) | Light intensity  (μmol m^-2^ s^-1^) | Spectral composition (μmol s^-1^) | | | | | Blue/Red |
| --- | --- | --- | --- | --- | --- | --- | --- | --- |
|  |  |  | Ultraviolet | Blue | Green | Red | Near  Infrared |  |
| TB | 97.04±2.45a | 36.0 | 0.07±0.01a | 9.33±0.59a | 17.41±1.08a | 9.20±0.53a | 0.82±0.05a | 1.01±0.01b |
| GB | 76.58±1.85b | 28.4 | 0.06±0.00b | 6.51±0.06c | 15.46±0.09a | 6.37±0.18b | 0.67±0.01b | 1.02±0.02b |
| BB | 52.01±3.75c | 19.2 | 0.04±0.00c | 8.02±0.23b | 8.17±0.32b | 3.05±0.14c | 0.39±0.01c | 2.63±0.05a |
